# Safety and Efficacy of 630-nm Red Light on Cognitive Function in Older Adults With Mild to Moderate Alzheimer’s Disease: Protocol for a Randomized Controlled Study

**DOI:** 10.3389/fnagi.2020.00143

**Published:** 2020-05-21

**Authors:** Nayan Huang, Dandan Yao, Wenjing Jiang, Cuibai Wei, Mo Li, Wenjie Li, Haiyan Mu, Maolong Gao, Zongjuan Ma, Jihui Lyu, Zhiqian Tong

**Affiliations:** ^1^Beijing Institute of Brain Disorders, Laboratory of Brain Disorders, Ministry of Science and Technology, Collaborative Innovation Center for Brain Disorders, Capital Medical University, Beijing, China; ^2^Center for Cognitive Disorders, Beijing Geriatric Hospital, Beijing, China; ^3^Innovation Center for Neurological Disorders, Xuan Wu Hospital, Capital Medical University, Beijing, China; ^4^Department of Neurology, Xuan Wu Hospital, Capital Medical University, Beijing, China; ^5^Institute for Geriatrics and Rehabilitation, Beijing Geriatric Hospital, Beijing, China

**Keywords:** Alzheimer’s disease, cognitive function, red light treatment, formaldehyde, functional magnetic resonance imaging

## Abstract

**Introduction:** Studies have shown that excess formaldehyde accumulation in the brain accelerates cognitive decline in people with Alzheimer’s disease (AD). Recently, reports from our research team revealed that red light treatment (RLT) improved memory in AD mice by activating formaldehyde dehydrogenase (FDH) and thus reducing formaldehyde levels. Here, we developed a medical RLT device to investigate the safety and efficacy of this device in older adults with mild to moderate AD.

**Methods:** This will be a randomized controlled trial (RCT) that will include 60 participants who will be recruited and randomly divided into an RLT group and a control group. The RLT group will receive RLT intervention 5 days a week for 30 min each time for 24 weeks while the control group will continue their routine treatments without RLT. All participants will undergo neuropsychological and functional assessments including the Mini-Mental State Examination, the AD assessment scale-cognitive subscale (ADAS-cog), the Geriatric Depression Scale (GDS), the Neuropsychiatric Inventory (NPI) and the Barthel Index at baseline, 12 weeks and 24 weeks. All participants will undergo functional magnetic resonance imaging (fMRI) scanning and blood/urine biomarkers tests at baseline and 24 weeks. The primary outcome will be the ADAS-cog score while the secondary outcomes will be the GDS and NPI scores. Adverse events will be recorded and treated when necessary. Both an intention-to-treat analysis and a per-protocol analysis will be performed to evaluate the safety and efficacy of RLT.

**Discussion:** This protocol outlines the objectives of the study and explained the RLT device developed by the research team. The study is designed as an RCT to evaluate the safety and effects of the RLT device on older adults with mild to moderate AD. This study will provide evidence for the clinical use of RLT on treatment for AD.

**Clinical Trial Registration:** www.ClinicalTrials.gov, ChiCTR1800020163; Pre-results.

## Introduction

As the most common cause of dementia in the elderly, Alzheimer’s disease (AD) is becoming increasingly prevalent as the population ages. The Alzheimer’s Association has now estimated and reported that the number of people with AD in the world will be more than 130 million by 2050 (Alzheimer’s Association, 2019). The neuropathology of AD is characterized by the accumulation of β-amyloid (Aβ)-related amyloid plaques and phosphorylated tau (p-tau)-related neurofibrillary tangles outside and inside neurons, respectively. These changes are widely believed pathogenic to the onset of AD (Serrano-Pozo et al., 2011; Alzheimer’s Association, 2019). As a persistent, disabling, and costly disease, AD unavoidably puts a huge burden on the family and society (Jia et al., 2018). Unfortunately, over the past decades new medications targeting Aβ production, aggregation and clearance, and tau hyperphosphorylation, such as antibodies, vaccines, and small molecule medicines, have not resulted in desirable clinical efficacy (Lane et al., 2018).

Recently, as a non-pharmacological therapy, low levels of laser light have been found to disaggregate Aβ (Son et al., 2018); hence phototherapy targeting Aβ is being increasingly investigated as an alternative therapy for AD (Salehpour et al., 2018). Previous studies showed that endogenous formaldehyde (FA) concentrations were gradually accumulated in animals and humans during the aging process and that FA was abnormally elevated in AD patients (Tulpule and Dringen, 2013). Indeed, excess FA injection led to memory decline in healthy animal models (Tong et al., 2013, 2017; Wang et al., 2019). Notably, excess FA also directly induces Aβ aggregation (Chen et al., 2006, 2007; Rizak et al., 2014; Yang et al., 2014; Liu et al., 2018), tau protein phosphorylation and aggregation (Lu et al., 2013; Yang et al., 2014; He et al., 2017; Liu et al., 2018), and oxidative stress (Songur et al., 2008; MacAllister et al., 2011). These findings strongly suggest that disaggregation of Aβ by scavenging FA may contribute to the treatment of AD. Our recent studies found that red light at 630 nm improved cognitive function by activating formaldehyde dehydrogenase (FDH) and thus degrading FA and increasing catalase activity to reduce oxidative stress in senescence-accelerated mouse-prone 8 (SAMP8) mice (Zhang et al., 2019). Red light at 630 nm also reversed memory deterioration by de-aggregating Aβ and enhancing FA metabolism in amyloid precursor protein/presenilin-1 (APP/PS1) transgenic AD mice (Yue et al., 2019).

Based on the above experimental evidence in animal models, our research team developed a therapeutic device for people with AD by using light-emitting diodes (LED) with a 630-nm red light in 2015. The penetration of the LED red light at 630 nm has been previously tested and we found that 630 nm red light had a strong penetration rate into the human brain cortex (approximately 48%) and liver (approximately 68%; Yue et al., 2019). This device is made of three main parts, including a power control board, an LED helmet for transcranial irradiation of the head, and an LED belly band for transabdominal illumination of the liver (Figure 3), and works by reducing the body’s FA levels by activating FDH in the brain and liver. In this study, a 24-week randomized controlled trial (RCT) was designed to investigate the safety and efficacy of this therapeutic red light treatment (RLT) device on older adults with mild to moderate AD. This protocol is reported according to the Standard Protocol Items: Recommendations for Intervention Trials (SPIRIT) guidelines (Chan et al., 2013).

**Figure 1:**
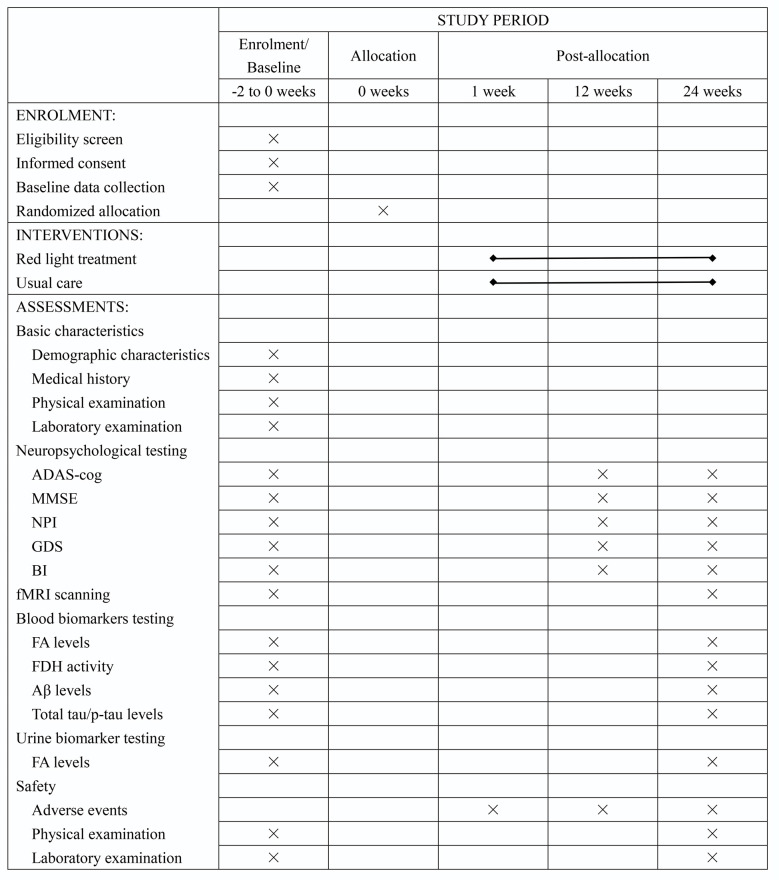
Schedule of recruitment, intervention, and assessment. Abbreviation: ADAS-cog, Alzheimer’s disease assessment scale-cognitive subscale; MMSE, Mini-Mental State Examination; NPI, Neuropsychiatric Inventory; GDS, Geriatric Depression Scale; BI, Barthel Index; fMRI, functional magnetic resonance imaging; FA, formaldehyde; FDH, formaldehyde dehydrogenase; Aβ, β-amyloid peptides; p-tau, phosphorylated tau protein.

**Figure 2:**
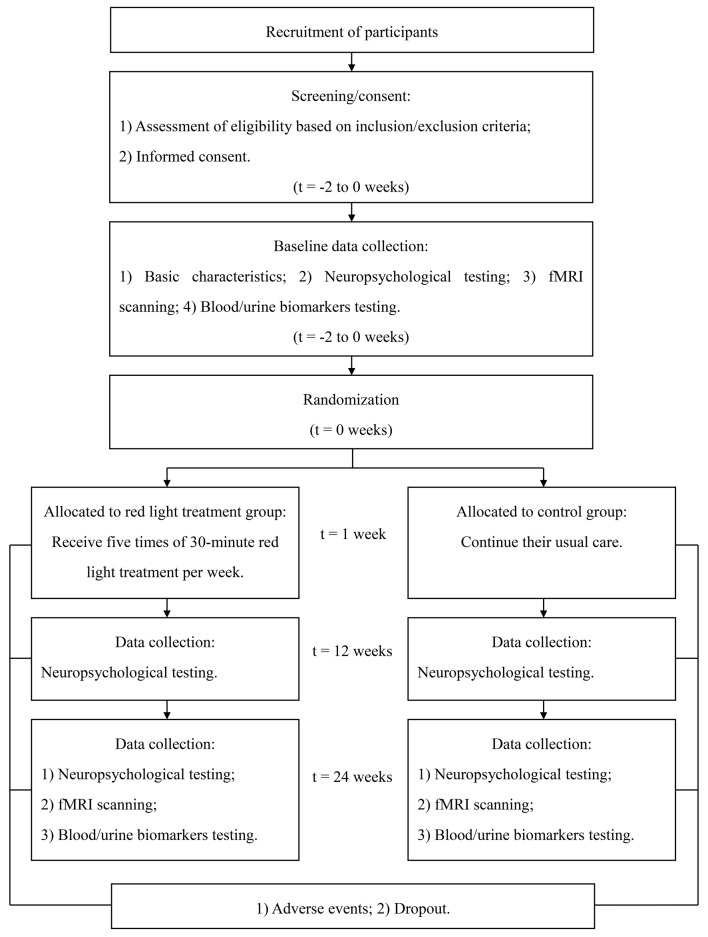
Flow diagram of study design. Abbreviation: fMRI, functional magnetic resonance imaging.

**Figure 3:**
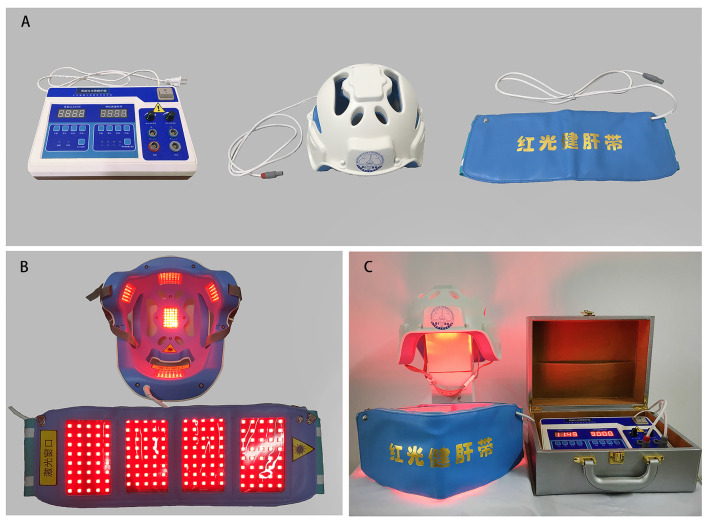
The therapeutic device: a 630-nm red light device used for the treatment of AD patients. **(A)** The device is constructed of three main parts. From left to right, there is a power control board, an LED helmet for transcranial illumination of the head, and an LED belly band for transabdominal irradiation of the liver. **(B,C)** Images of the therapeutic illuminated LED lights in the “ON” position for treatment of AD patients. Abbreviation: LED, light-emitting diode.

### Objectives

The primary objectives are to test the safety and effects of the RLT on cognitive function, behavior/mood, and activities of daily living in older adults with mild to moderate AD.

The secondary objectives are: (1) to explore the changes in brain function by using functional magnetic resonance imaging (fMRI); and (2) to examine the changes in the levels or activities of blood/urine biomarkers, such as FA, Aβ, total tau, p-tau, and FDH.

## Methods

### Settings

This device was developed and this study was designed at the Beijing Institute of Brain Disorders, Capital Medical University, China. The recruitment, assessment, intervention, and follow-ups of participants will be conducted at the Center for Cognitive Disorders of Beijing Geriatric Hospital (BGH), China. The Case Report Forms will be filled and kept in BGH.

### Study Design

This is an RCT study with two parallel arms (1:1 allocation ratio): an intervention group receiving an RLT program and a control group receiving routine therapy without RLT treatment (Figures 1, 2).

### Recruitment of Participants

Participants will be recruited from the clinic, inpatient ward, or long-term care facilities near BGH. Residents who meet the inclusion criteria will be recruited.

### Inclusion Criteria

The inclusion criteria will be as follows: (1) 60 years old or older; (2) with a diagnosis of probable AD based on the National Institute of Neurological and Communicative Disorders and Stroke and the AD and Related Disorders Association (NINCDS-ADRDA) criteria (McKhann et al., 1984); and (3) with a clinical dementia rating score ≤2. If the participants are receiving cholinesterase inhibitors, memantine, or other dementia-related medications, they will have to take a therapeutic and stable dose for at least 3 months before screening.

### Exclusion Criteria

Participants will be excluded if they are experiencing any of the following conditions: (1) severe visual or auditory impairment; (2) serious medical conditions in major organs (such as the heart, lung, kidney, or liver) that limit the ability to participate in the study; (3) current alcohol or drug abuse; (4) any other conditions which may disturb assessments or interventions required in this study; (5) history of epilepsy; or (6) having been enrolled into any other interventional studies.

### Randomization and Allocation

Participants will be enrolled by dementia specialists. The randomization will be carried out by an independent research assistant who will not be involved in the enrollment, assessment, or intervention of the participants. Random number sequences will be generated using SAS software version 9.4 (SAS Institute Inc., Cary, NC, USA). Sealed envelopes with the serial number outside and group number inside will be produced and kept in a locked drawer that will be inaccessible to all the researchers. The envelopes will be opened sequentially by the independent research assistant after baseline assessments, and participants will be assigned to an intervention group and a control group at a ratio of 1:1 according to the group number printed inside the envelopes. Outcome evaluators and data analysts will be blinded to the group assignment.

### Interventions

In addition to their routine treatments and personalized daily care, participants in the RLT group will receive the 24-week intervention of RLT with a frequency of 5 days per week for 30 min each time. The RLT treatment will be performed by researchers or caregivers who are trained to operate the RLT device in advance. The participants will be put on the LED helmet and the LED belly band and receive RLT therapy with concurrent transcranial and transabdominal illumination in the morning (Figure 3). The light parameters of the RLT device are shown in Table 1. The researchers or caregivers will be asked to keep a strict watch over the participants during the intervention process. A form of RLT treatment schedule will be made for each participant in the RLT group. The researchers or caregivers who perform the RLT intervention will be asked to sign on the report form after every session of treatment to ensure compliance of the intervention.

**Table 1:** Parameters of the therapeutic device of 630-nm red light for AD patients.

Source	The red light treatment device
Wavelength (nm)	630 ± 15
Power output per LED (mW)	5
Power density (mW/cm^2^)	20 (anterior helmet LEDs)
	40 (posterior helmet LEDs)
	5 (temporal helmet LEDs)
	5 (belly band LEDs)
Duration of each time (min)	30
Frequency of treatment	5 times per week

*Abbreviation: AD, Alzheimer’s disease; LED, light-emitting diode*.

Participants in the control group will continue their routine treatments and personalized daily care during the 24-week study period, including usual medicine, recreational therapy, and management of behavioral disorders following current guidelines. Except for the RLT intervention for the RLT group, all the other treatment strategies for the two groups will be the same.

### Demographic Data Collection

Demographic characteristics of all participants including age, gender, educational level, as well as medical history, medication list, and comorbidities will be documented.

### Neuropsychological Assessments

A set of neuropsychological tests will be used to assess cognitive function and behavior/mood of all the participants, and their activities of daily living will also be measured. The assessments will be performed at baseline, 12 weeks, and 24 weeks during the study period. (1) The Mini-Mental State Examination (Katzman et al., 1988) is the most frequently used assessment to measure global cognitive function. It is a 30-point scale that assesses orientation, memory, attention and calculation, recall, and language, with higher scores indicating better cognitive function. (2) The AD assessment scale-cognitive subscale (ADAS-cog; Chu et al., 2000) is a 12-item scale that primarily measures word recall, ability to follow commands, constructional praxis, naming, ideational praxis, orientation, word recognition, comprehension of spoken language, word-finding, language ability, and attention. The scores range from 0 to 75 and higher scores indicate greater cognitive impairment. (3) The Geriatric Depression Scale (GDS; Yesavage et al., 1982) is a short questionnaire to identify depression in older people and it is validated in mild to moderate dementia. (4) The Neuropsychiatric Inventory (NPI; Wang et al., 2012) is an assessment of 12 different behavioral and psychological symptoms common in AD. Each symptom is rated and scored according to the frequency and severity reported by the informants. The distress level of the caregivers associated with each symptom is also rated. (5) The Barthel Index (Mahoney and Barthel, 1965), is a 10-item, ordinal scale used to measure performance in activities of daily living. It is also performed by interviewing the caregivers.

### fMRI

fMRI is a non-invasive brain imaging technique to measure and map brain activity. In this study, we will collect the data of resting-state fMRI (rs-fMRI) for the analysis of neurophysiological changes (Lee et al., 2013; Smitha et al., 2017). Participants will receive fMRI scans by using a Philips Ingenia 3.0 T magnetic resonance system at BGH at baseline and 24 weeks. The following sequences will be acquired: (1) High-resolution anatomic images using a 3D T1-weighted inversion recovery turbo field echo sequence with the following parameters: repetition time (TR) 7.9 ms, echo time (TE) 3.5 ms, contiguous 180 sagittal slices of 1 mm thickness, flip angle 8°, 1 × 1 × 1 mm voxels, 160 × 210 × 180 matrices, and field of view (FOV) 160 × 211 × 180 mm. (2) Single-shot field echo echo-planar imaging sequence for rs-fMRI images with the following parameters: 200 sets of contiguous 40 transverse slices, 4 mm thickness, TR 3,000 ms, TE 35 ms, flip angle 90°, 2 × 2 × 4 mm voxels, 120 × 116 × 40 matrices, and FOV 240 × 240 × 160 mm. The total acquisition time of rs-fMRI is 609 s. During the scanning process, participants will be asked to keep their eyes closed and remain motionless.

### Blood/Urine Biomarkers

The biomarkers of blood and/or urine of all participants will be measured at baseline and 24 weeks. The concentrations of blood Aβ, total tau, and p-tau, which are related to the pathology of AD, will be quantified by human Aβ and tau ELISA kits. Meanwhile, blood and urine FA levels and blood FDH activity will be measured.

### Outcomes

The primary outcome will be the ADAS-cog score. The secondary outcomes will be the GDS and NPI scores. The safety of the RLT will be evaluated according to the incidence and severity of adverse events such as headache, dizziness, or nausea. The adverse events will be immediately reported, and handled by experienced physicians. Details of these possible adverse events and the treatments they receive will be recorded by filling in the case report forms. Physical examinations and necessary laboratory examinations, such as blood routine examination, liver function, and renal function, will be completed at baseline and the end of this study.

### Sample Size Calculation

No studies have investigated the effects of RLT treatment on people with AD. However, there have been relevant published literature that reported positive effects of photobiomodulation (PBM) with 810 nm near-infrared light on people with dementia. For example, PBM was shown to reduce ADAS-cog scores by 6.73 points after a 12-week consecutive treatment (Saltmarche et al., 2017), and by 5.18 points in another report (Chao, 2019). In this study, we anticipate at least an effect size of 0.48 in ADAS-cog scores after 24-week RLT intervention. The sample size was calculated using Power Analysis and Sample Size software version 15.0 (NCSS, LLC)^1^. A total sample size of 48 participants is sufficient to detect the target effect size with 90% power (*β* = 0.10) and a type I error of 5% (*α* = 0.05). Considering a dropout rate of 10%, a total of 54 participants is necessary. In this study, we intend to recruit 60 participants, with 30 participants in each group.

### Quality Control and Quality Assurance

At least three dementia specialists will work together to examine the participants and provide a diagnosis for each participant. All data will be monitored and reviewed by the principal investigator or research coordinators. Training will be provided to all researchers. Consistency coefficients in scoring assessment scales between researchers should be no less than 0.85. Data entry will be verified by a second researcher in the team. To protect participant confidentiality, only supervisors, researchers of this study, and the ethics committee will be authorized to access to the personal information and medical records of the participants.

### Statistical Methods

Statistical analysis of demographics and clinical characteristics will be performed using IBM SPSS Statistics for Windows, Version 20.0 (Release 2011, IBM Corp, Armonk, NY, USA). Continuous data will be reported as means ± standard deviations or 95% confidence intervals, and as numbers with percentage for categorical data. The difference in demographic and baseline characteristics between two groups will be analyzed by using the independent *t*-test, and a *chi*-square test will be used for categorical variables.

The main analysis method for the safety and efficacy of RLT intervention in this study will use the intention-to-treat analysis including all randomized participants (Hollis and Campbell, 1999). Besides, a per-protocol analysis will be performed on individuals who complete the total study (Sedgwick, 2015). The repeated measures of variance analysis will be used to examine variations of scores of neuropsychological tests over time between two groups. The association between intervention and incidence of adverse events will be analyzed using the *chi*-square test.

fMRI data will be preprocessed by using Statistical Parametric Mapping version 12.0 (The Wellcome Trust Centre for Neuroimaging at UCL, London, UK) on the MATLAB platform (Release 2013b, The MathWorks Inc, Natick, MA, USA). There are several critical steps including motion correction, slice timing correction, spatial normalization, and smoothing. After fMRI data preprocessing, the fractional amplitude of low-frequency fluctuation and functional connectivity analysis will be analyzed by using the REST software version 1.8 (Song et al., 2011).

The level of significance for statistical analysis will be set at 5% (*P* < 0.05). The researchers who perform the data analysis will be blinded to the allocation and the intervention.

### Patient and Public Involvement

The original research question and outcome measures were conceived by the authors and were then modified based on face-to-face screening interviews with 16 elderly volunteers with cognitive impairment by a research assistant. They were also invited to use the RLT device for 3 months during the design phase of this study. The duration of treatment per time and frequency of treatment was determined based on their feedback to ensure the tolerance and the applicability of the intervention. These volunteers will not be included in this study. Both the potential burden and benefit of this study will be assessed by the participants and their guardians before signing the informed consent. The findings of this study will be made available to the participants and their guardians.

## Ethics and Dissemination

This study protocol has been approved by the ethical review committee of the Beijing Geriatric Hospital (approval number: 2019-024). Information leaflets of the study will be available at the public area of the Center for Cognitive disorders of BGH. The leaflets will explain in full detail the aims and objectives of the study, selection criteria, and the processes that the study will be adhering to. The research team will provide an individual face to face consultation to all potential participants and their guardians to answer any questions they may have before signing the consent form. Informed consent will be obtained from all participants and/or their legal representatives. Participants will be allowed to withdraw from the study at any time and the reason for withdrawal will be recorded. The results of this study will be reported in peer-reviewed journals and presented at national or international conferences on aging and dementia.

## Discussion

To our knowledge, this is the first RCT to investigate the safety and efficacy of RLT on AD. This 24-week study is designed to evaluate the effects of RLT intervention on cognitive function, mood, psychiatric and behavioral performances, and activities of daily living in older adults with mild to moderate AD. Also, neurophysiological changes assessed by fMRI and changes in blood/urine biomarkers associated with RLT will also be evaluated.

Preliminary evidence for the benefits of the RLT on AD has been obtained. The RLT can not only prevent early-stage memory decline but also rescue late-stage memory deficits in AD mice by reducing FA levels and oxidative stress (Zhang et al., 2019). Moreover, PBM with red to near-infrared light has been proposed as a non-invasive and innovative therapy for brain disorders. Some other beneficial effects of PBM have been reported, such as improved cerebral metabolic function (Oron et al., 2007; Rojas et al., 2012; Ferraresi et al., 2015), increased expression of neurotrophic factor expression, improved neurogenesis (Oron et al., 2006; Xuan et al., 2014, 2015), and reduced neuroinflammation (Lee et al., 2016; Aragona et al., 2017). Therefore, PBM appears to be a promising strategy for AD treatment.

There are limitations to this study. The first is that as the first preliminary clinical trial for the RLT device it is a single-center design. The second is the absence of a sham intervention for the control group for ethical and practical considerations. To minimize these shortcomings, the processes of randomization and blindness will be strictly carried out, and objective examinations including fMRI and blood/urine biomarkers will be conducted. Moreover, because of the cognitive impairments in patients with AD, the placebo effect can be greatly eliminated. The third is that follow-up assessment after cessation of the intervention is not planed in this protocol because the effects of the RLT are still unknown. If positive results are found in this initial study, future research will be conducted to assess the long-term influence of the RLT, which will help to identify the optimal RLT duration.

In summary, this RCT will explore the potential beneficial effects and safety of RLT on older adults with mild to moderate AD. The study will provide evidence for the clinical use of RLT on treatment for mild to moderate AD.

## Ethics Statement

The studies involving human participants were reviewed and approved by the Medical Ethics Committee of the Beijing Geriatric Hospital (approval number:2019-024). The patients/participants provided their written informed consent to participate in this study.

## Author Contributions

ZT and JL conceived and designed the study. All authors played a role in the development of the protocol. NH drafted the manuscript, and all authors participated in the preparation of this manuscript. All authors approved the submission of this manuscript.

## Conflict of Interest

The authors declare that the research was conducted in the absence of any commercial or financial relationships that could be construed as a potential conflict of interest.

## Footnotes

^1^https://www.ncss.com/
